# Supplementary material for: Accurate Bayesian phylogenetic point estimation using a tree distribution parameterized by clade probabilities
Source: PLoS Comput Biol. 2025 Feb 13;21(2):e1012789. doi: 10.1371/journal.pcbi.1012789 (PMC11835378; doi:10.1371/journal.pcbi.1012789)
Supplement: S1 Text — Containing 4 supporting sections describing further details and results, 15 additional figures, and a table. (PDF) [file pcbi.1012789.s001.pdf]

Supporting Information

S1.1 Additional Details on Methods

General Parametrizations of CCD0. We now prove the statement from Section 2.1 that observed samples frequencies can be converted into clade split probabilities of a CCD. In fact, we show a more general result, namely, that we can turn a forest network into a CCD based on a clade scoring function $F\colon 2^X \to [0,1]$ with $F(\{\ell\}) = F(X) = 1$ where 2^X is the power set of X and $\ell \in X$. For CCD0, this function would be $F(C) = \Pr'(C)$, i.e. the Monte Carlo probability of C in the sample. For a tree T on X, define $F(T) = \prod_{C \in \mathcal{C}(T)} F(C)$, i.e., the product for the clades of T. Let $\mathcal{C}$ be the subset of 2^X containing all clades with non-zero scores ($F(C) > 0$). Let $\mathcal{S}$ be the set of clade splits that should be in the CCD. This could be the observed clade splits as in CCD1 or all possible clade splits that can be formed from $\mathcal{C}$ as in CCD0. Let G be the forest network based on $\mathcal{C}$ and $\mathcal{S}$.

For a clade C and forest network G, recall that $G(C)$ is the set of all trees in G rooted at C. Let $F_+\colon \mathcal{C} \to \mathbb{R}$ be the function defined as $F_+(C) = \sum_{T \in G(C)} F(T)$. This can be computed recursively with the formula

$$F_+(C) = F(C) \cdot \sum_{\{C_1, C_2\} \in \mathcal{S}(C)} F_+(C_1) F_+(C_2)$$

with base case $F_+(\{\ell\}) = 1$ for each $\ell \in X$. Note that $F_+(X)$ is the sum over all trees on X and hence $\alpha = 1/F_+(X)$ is the global normalization factor turning the score $F(T)$ of a tree T into a probability $\Pr(T) = \alpha F(T)$. Populating the clades in G with F, we can compute $F_+(X)$ efficiently (more precisely, fixed-parameter tractable in the number of clades and clade splits with non-zero scores). Moreover, we show next that we can also define probability distributions for the clade splits and hence turn G into a CCD graph.

For a clade C, with clade splits $\mathcal{S}(C)$, define the normalization factor α_C for C as $\alpha_C = 1/\sum_{\{C_1, C_2\} \in \mathcal{S}(C)} F_+(C_1) F_+(C_2)$ and thus $\alpha_C F_+(C) = F(C)$. Then the probability of a clade split $\{C_1, C_2\}$ of C is $\Pr(\{C_1, C_2\}) = \alpha_C F_+(C_1) F_+(C_2)$. We have to show that this gives the same probability for a tree T:

$$\begin{aligned}
\Pr(T) &= \prod_{\{C_1, C_2\} \in \mathcal{S}(T)} \Pr(\{C_1, C_2\}) \\
&= \prod_{\substack{\{C_1, C_2\} \in \mathcal{S}(T) \\ C_1 \cup C_2 = C}} \alpha_C F_+(C_1) F_+(C_2) \\
&\overset{(1)}{=} \prod_{\substack{\{C_1, C_2\} \in \mathcal{S}(T) \\ C_1 \cup C_2 = C}} \frac{\alpha_C F(C_1) F(C_2)}{\alpha_{C_1} \alpha_{C_2}} \\
&\overset{(2)}{=} \alpha \prod_{C \in \mathcal{C}(T)} F(C) = \alpha F(T)
\end{aligned}$$

where for (1) we use that $F_{+}(C) = F(C)/\alpha_C$, and for (2) observe that $\alpha = \alpha_X$, that all other α_C are 1 for singleton clades or cancel out (they appear once in the denominator where C is part of the clade split and once in the nominator for the clade split of C). Hence, using $F(C) = \Pr'(C)$, we get that G is a CCD graph as desired, our new tree distribution CCD0.

CCD1 and Monte Carlo Clade Probabilities. Recall that for a tree (multi-)set $\mathcal{T} = \{T_1, \ldots, T_k\}$ and a clade C (clade split S), the frequency $f(C)$ (resp. $f(S)$) is how often C (resp. S) appears in $\mathcal{T}$. The Monte Carlo probability of C is then $f(C)/k$. We show that, for a CCD1 with parameters populated by the frequencies in $\mathcal{T}$, the probability $\Pr(C)$ of a clade appearing in a random tree of the CCD1 is equal to its Monte Carlo probability in $\mathcal{T}$. Note that the probability of the root clade C_ρ is one in both the CCD1 and the sample. Assume now that the statement holds for all parent clades $C_1, \ldots, C_\ell$ of a clade C. For $i \in \{1, \ldots, \ell\}$, let S_i be the clade split of C_i containing C. Note that $\sum_{i=1}^{\ell} f(S_i) = f(C)$. We can now sum over the product of probabilities of the parent clades and their respective clade splits to get the probability of C, which simplifies to the desired Monte Carlo probability:

$$\Pr(C) = \sum_{i=1}^{\ell} \Pr(C_i) \Pr(S_i) = \sum_{i=1}^{\ell} \frac{f(C_i)}{k} \frac{f(S_i)}{f(C_i)} = \sum_{i=1}^{\ell} \frac{f(S_i)}{k} = \frac{f(C)}{k}$$

S1.2 Graphical Models of Datasets

```
data {
   L = 300;
   clockRate = 1.0;
   nCatGamma = 4;
   birthRate = 25.0;
   n = 50;
}
model {
   frequencies ~ Dirichlet(conc=[5.0, 5.0, 5.0, 5.0]);
   kappa ~ LogNormal(meanlog=1.0, sdlog=1.25);
   Q = hky(kappa=kappa, freq=frequencies);
   shape ~ LogNormal(meanlog=-1.0, sdlog=0.5);
   siteRates ~ DiscretizeGamma(shape=shape, ncat=nCatGamma, replicates=L);
   phi ~ Yule(lambda=birthRate, n=n);
   D ~ PhyloCTMC(L=L, Q=Q, mu=clockRate, siteRates=siteRates, tree=phi);
}
```

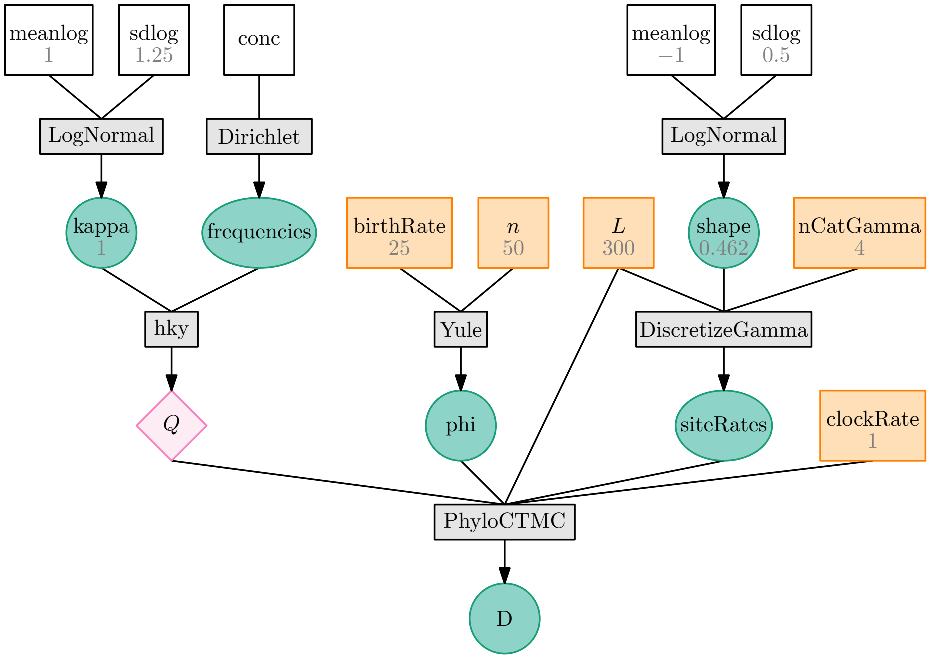

Fig A: lphy script and graphical model of Yule datasets, here for $n = 50$.

```
data {
   L = 250;
   clockRate = 1.0;
   nCatGamma = 4;
   n = 40;
}
model {
   frequencies ~ Dirichlet(conc=[5.0, 5.0, 5.0, 5.0]);
   kappa ~ LogNormal(meanlog=1.0, sdlog=1.25);
   Q = hky(kappa=kappa, freq=frequencies);
   shape ~ LogNormal(meanlog=-1.0, sdlog=0.5);
   siteRates ~ DiscretizeGamma(shape=shape, ncat=nCatGamma, replicates=L);
   positiveAges ~ Uniform(lower=0, upper=0.2, replicates=n-1);
   leafAges = concatArray([0.0], positiveAges);
   popSize ~ LogNormal(meanlog=-2.4276, sdlog=0.5);
   phi ~ Coalescent(ages=leafAges, n=n, theta=popSize);
   D ~ PhyloCTMC(L=L, Q=Q, mu=clockRate, siteRates=siteRates, tree=phi);
}
```

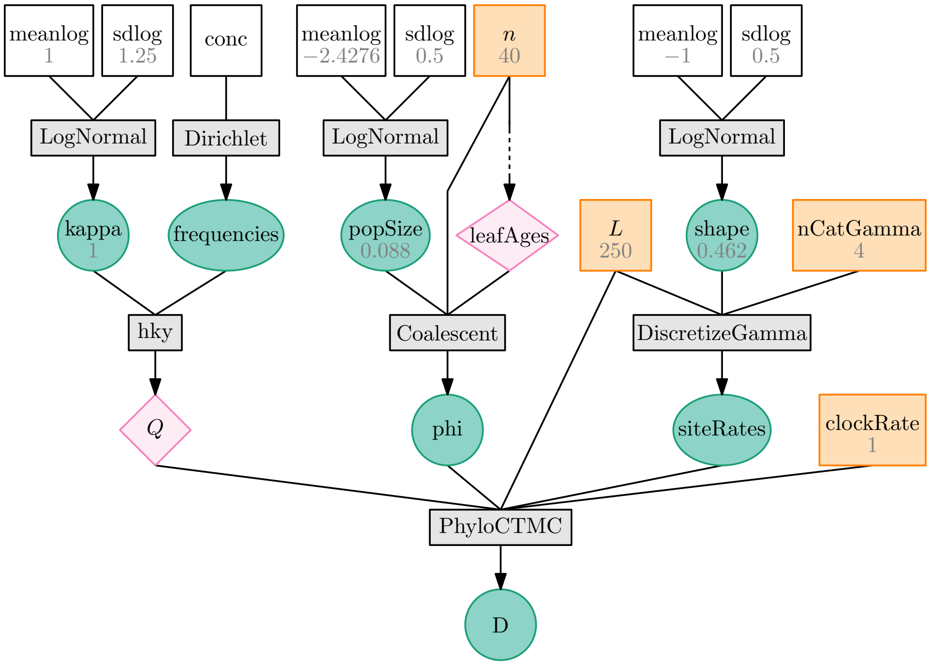

Fig B: lphy script and graphical model of Coalescent datasets, here for $n = 40$

S1.3 Further Results on the Distributions

S1.3.1 Yule10 and Yule20

The mean MAE values for different entropy categories and the different methods are shown in Fig C. The "wins" of the different methods in Fig 4 are based on these MAE values. We also present the distribution of "wins" for the top and bottom row of the heatmap Fig 8, that is the 50 simulations with highest and lowest entropies.

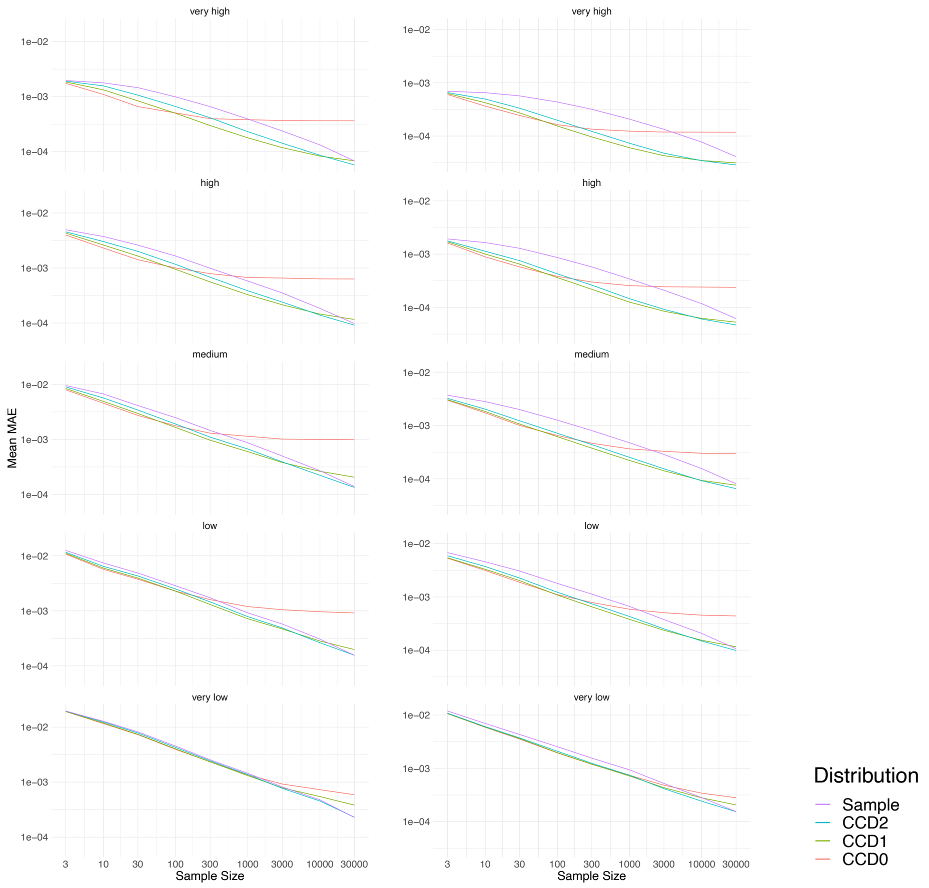

(a) `Yule10` (b) `Yule20`

Fig C: Mean Mean Absolute Error (MAE) of the different distributions with simulations in five entropy categories (higher means noisier/harder).

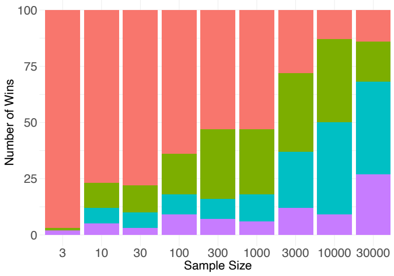

(a) `Yule 10`, 50 lowest entropy simulations

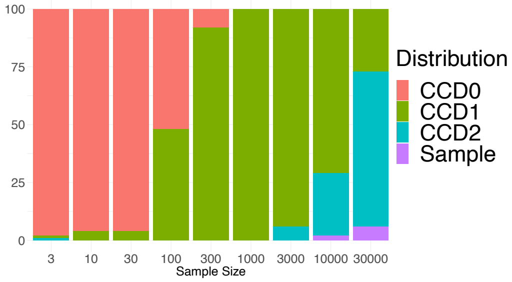

(b) `Yule 10`, 50 highest entropy simulations

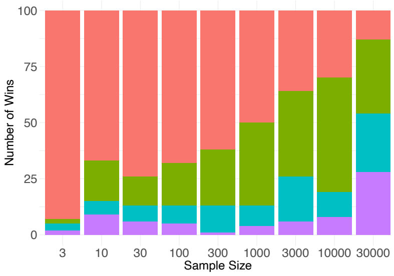

(c) `Yule 20`, 50 lowest entropy simulations

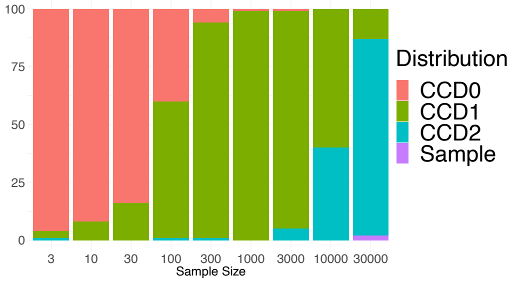

(d) `Yule 20`, 50 highest entropy simulations

Fig D: For 50 simulations of a particular entropy group, two replicates each and a sample size, the number of bars in one color represent how often that distribution had the lowest MAE.

The median MRE of clade and of tree probabilities of the trees in the 50% and 95% credibility intervals for `Yule10` are shown in Figure E showing the same results as for `Yule20` in Fig 5.

Figure F shows the mean estimated rank of the top tree of the golden distribution in the other distributions for `Yule10`. One difference to the `Yule20` results in Fig 6 is that the difference between the sample distribution (CCD1) and CCD0 is smaller (resp. larger) for sample sizes up to 300 trees, but overall the same tendencies emerge.

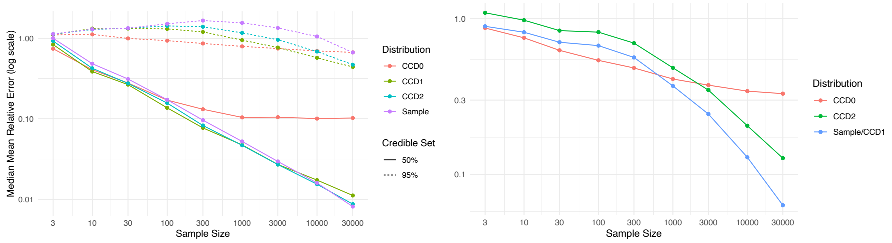

(a) Median MRE of trees. (b) Median MRE of clades.

Fig E: Median MRE for trees and clades in the golden distribution per sample size for `Yule10`. Trees are separated into the 50% and 90% credible sets.

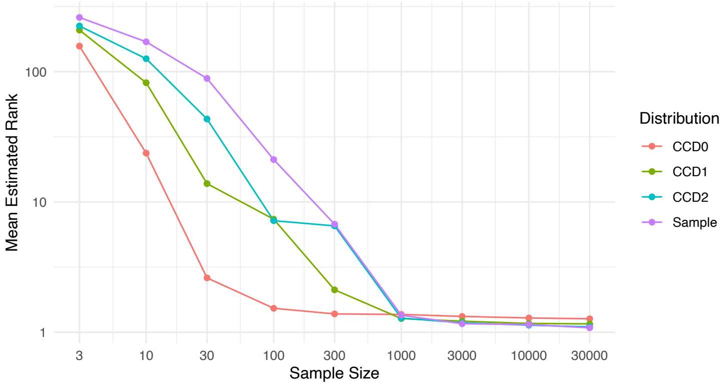

Fig F: Mean rank of the top tree (rank 1) in the golden distribution in the other distributions per sample size for `Yule10`.

S1.3.2 DS1 to DS4

The datasets `DS1` – `DS4` are popular benchmarking datasets in phylogenetics [14, 17, 28, 32] and have shown to have complex posterior tree distributions, unlike our well calibrated simulations `Yule10` and `Yule20`. For each of the datasets, we ran two replicates with BEAST2 to obtain tree samples with 1 million trees (after discarding sufficient burnin, following Whidden and Matsen [32]). For each simulation, we combined the 1 million trees from the two replicates into one sample distribution of 2 million trees, which acts again as our *golden distribution*. Even though these datasets have higher number of taxa (27 to 41), they all have entropies similar to the very high entropy category of `Yule20` simulations (between 5 and 6, cf. Fig 4). In `DS1`, for example, one tree was sampled over 11%. This is also the reason we only included four datasets, as for DS5–DS11 we could mostly not sample enough trees to have an accurate golden distribution (cf. Section 3.1).

We again used samples of size 3, 10, 30, 100, 300, 1k, 3k, 10k, and 30k to generate a CCD0, CCD1, CCD2 and a sample distribution for each of the two replicates of all simulations – eight distributions per simulation. For each tree T in the golden distribution, we then calculated the probability of T in each of the eight distributions and computed the absolute errors compared to the golden distribution. The results are shown in Figs G to J.

For `DS1`, we observe that the bias of each CCD becomes apparent and the richer models quickly overtake the simpler models; CCD1 performs better than CCD0 for a sample size of 10, then CCD2 becomes better than CCD1 for 30 trees and more, and finally the sample distribution offers the best estimate from 3k sampled trees on. So here, the richer model can better capture the structure of the posterior distribution. On the other hand, for `DS2` and `DS3`, the performances of the CCDs are hard to distinguish. This suggests that here the independence assumptions of the CCDs mostly hold and the richer model of CCD1 and CCD2 do not add much benefit. For `DS4`, both CCD1 and CCD2 seem to capture more structure than CCD0. The sample distribution requires large number of trees to catch up, for `DS2` to `DS3`.

We also calculated the Akaike information criterion (AIC) scores of the CCDs as follows. With k being the number of parameters and $\hat{L}$ the likelihood, the AIC score is $2k - 2\ln(\hat{L})$. The number of parameters k is the number of clades for CCD0 and the number of clade splits for CCD1 and CCD2. (Note that in a CCD2, the same clade splits can appear under multiple clades distinguished by their sibling clade, so their number is always at least the number of clade splits in a CCD1.) We computed the likelihood $\hat{L}$ as the product of the probabilities of the trees in the sample used to construct the CCDs. Since we subsampled from longer chains (35k trees), we may assume that the used trees are independent only for small sample sizes. For larger sample sizes, however, there may be a bias towards more complex models. The scores are shown in Figs G to J. For all four datasets, the AIC scores reflect the accuracy results with the difference that the change of preferred model happens at higher sample sizes.

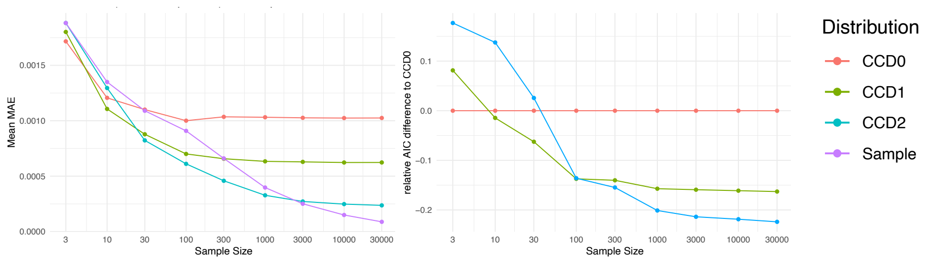

(a) Mean MAE (b) AIC relative to CCD0

Distribution	Sample Size 3	10	30	100	300	1k	3k	10k	30k
CCD0	**77.5**	192.7	447	1460	4322	13791	41k	138k	411k
CCD1	83.8	**189.9**	**419**	**1259**	3716	11624	35k	116k	344k
CCD2	91.2	219.2	459	1261	**3654**	**11018**	**33k**	**108k**	**319k**

(c) Absolute AIC values (rounded)

Fig G: DS1

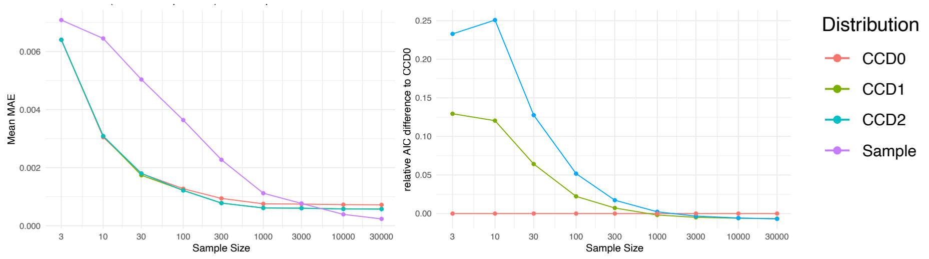

(a) Mean MAE (b) AIC relative to CCD0

Distribution	Sample Size 3	10	30	100	300	1k	3k	10k	30k
CCD0	**77.3**	**161.1**	**350**	**1103**	**3189**	10613	31607	104925	315349
CCD1	87.3	180.5	372	1128	3212	**10594**	**31453**	**104292**	313300
CCD2	95.3	201.5	395	1160	3245	10638	31504	104325	**313176**

(c) Absolute AIC values (rounded)

Fig H: DS2

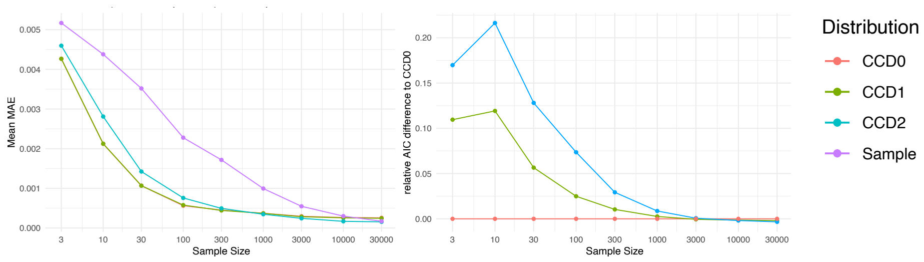

(a) Mean MAE (b) AIC relative to CCD0

Distribution	Sample Size 3	10	30	100	300	1k	3k	10k	30k
CCD0	**91.3**	**167**	**353**	**1067**	**3149**	**10215**	30334	101495	304961
CCD1	101.3	187	373	1094	3182	10242	**30317**	101349	304345
CCD2	106.8	204	399	1145	3242	10303	30355	**101305**	**303951**

(c) Absolute AIC values (rounded)

Fig I: `DS3`

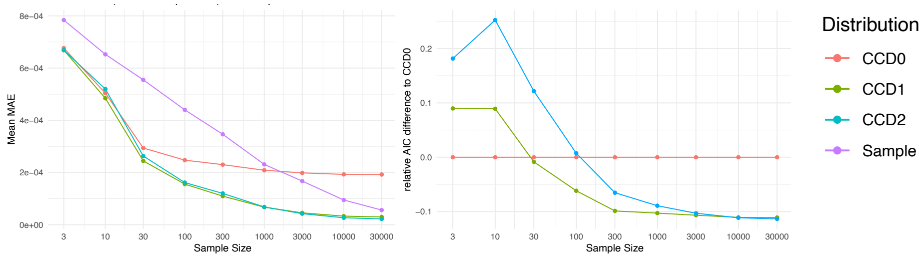

(a) Mean MAE (b) AIC relative to CCD0

Distribution	Sample Size 3	10	30	100	300	1k	3k	10k	30k
CCD0	**98.0**	**185**	492	1347	4035	13214	39989	132058	396881
CCD1	106.8	201	**488**	**1264**	**3636**	**11855**	**35721**	**117433**	352818
CCD2	115.8	231	552	1357	3772	12035	35866	117344	**351838**

(c) Absolute AIC values (rounded)

Fig J: `DS4`

S1.4 Further Results on Point Estimators

For completeness, we provide here the accuracy and precision results of the point estimators for the smaller datasets `Yule50`, `Yule100`, `Coal40`, and `Coal80`. The accuracy results are shown in Fig K (cf. Fig 8) and the precision results are shown in Fig L (cf. Fig 9). Furthermore, to highlight the improved precision of the CCD0-MAP tree over the MCC tree, we plotted 14 point estimates on 5k trees each for one of the `Coal160` datasets with DensiTree [36] in Fig M.

In addition, we also computed the stability of the point estimates, that is, the mean difference between the distances to the true tree between two corresponding replicates; see Fig N. We observe that the CCD-MAPs and greedy consensus are more stable than MCC, which is not surprising given their higher precision.

We further compared point estimates on the `Yule400` simulations when the number of sites increases. Specifically, we have added the `Yule400-Long` simulation whose alignment is 4 times longer, now containing 1200 sites. We evaluated the point estimators based on accuracy, stability, and precision. As expected, we observe in Fig O that longer alignments shift everything closer to the truth.

Table A: Number of unresolved greedy consensus trees out of 200 per dataset and sample size.

	Sample Size								
Dataset	3	10	30	100	300	1k	3k	10k	30k
`Yule50`	13	3	1	0	0	0	0	0	0
`Yule100`	24	8	1	0	0	0	0	0	0
`Yule200`	40	6	3	0	0	0	0	0	0
`Yule400`	59	21	5	1	0	0	0	0	0
`Coal40`	3	0	0	0	0	0	0	0	0
`Coal80`	23	3	0	0	0	0	0	0	0
`Coal160`	76	31	9	0	0	0	0	0	0
`Coal320`	162	92	9	2	0	0	0	0	0

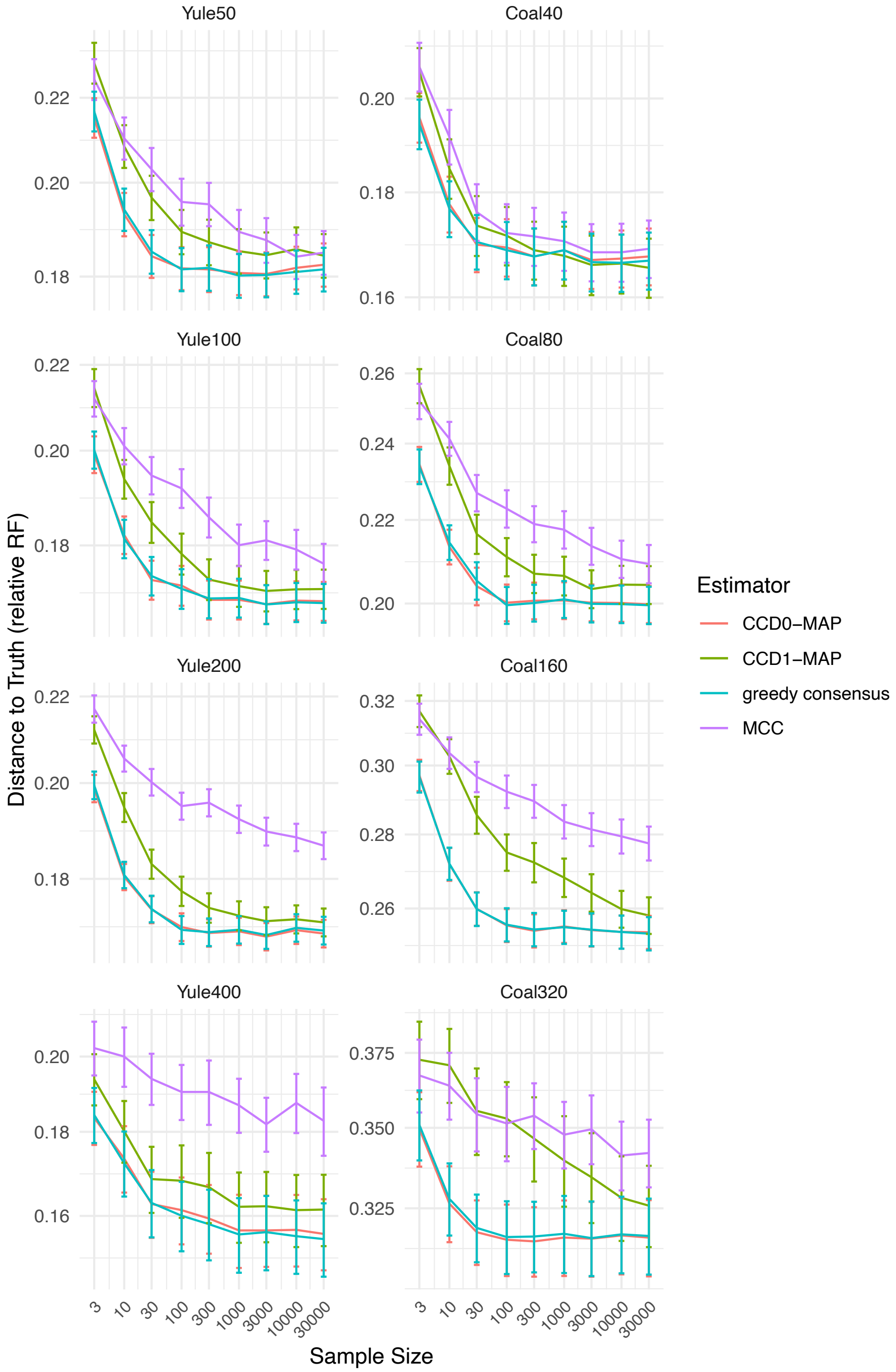

Fig K: The accuracy of the point estimates measured in terms of the mean relative RF distance to the true tree for different sample sizes of the large datasets.

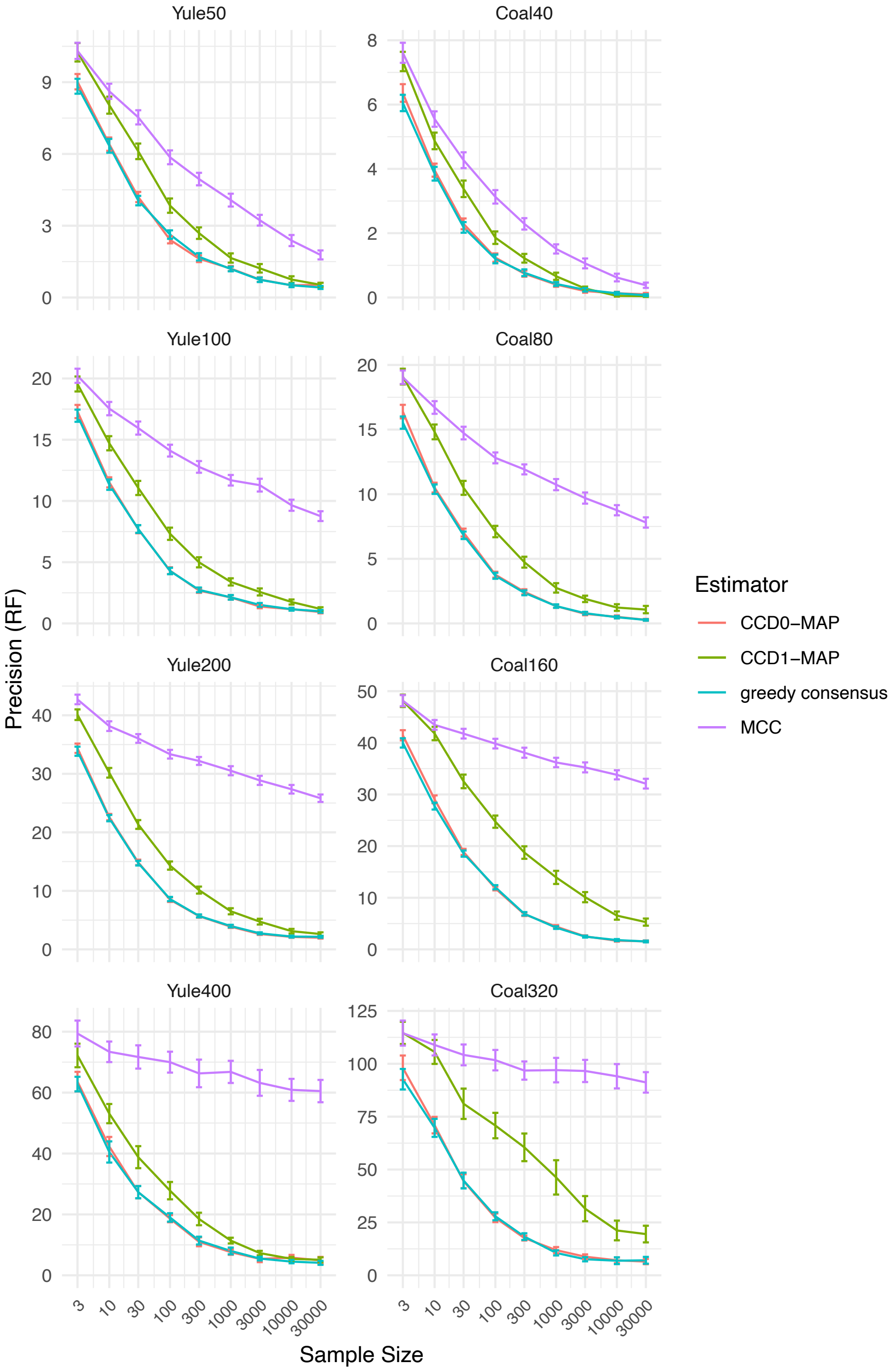

Fig L: The precision of the point estimates in terms of the RF distance, that is, the mean RF distance of the point estimates of the two replicates of each simulation.

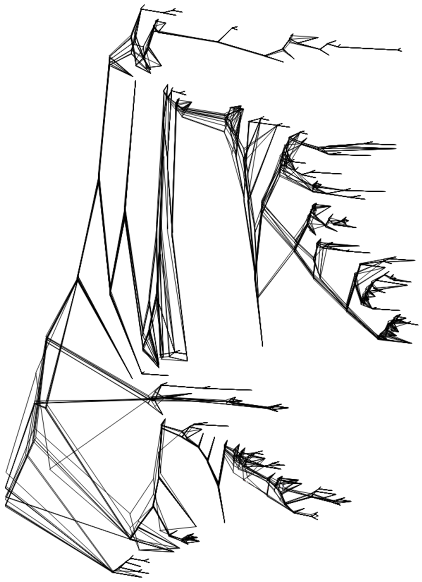

(a) MCC tree

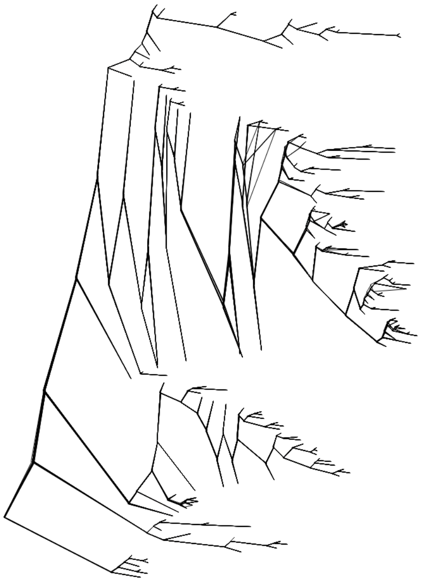

(b) CCD0-MAP tree

Fig M: Cloudograms from DensiTree of 14 point estimates on subsamples of 5k trees from one `Coal160` dataset.

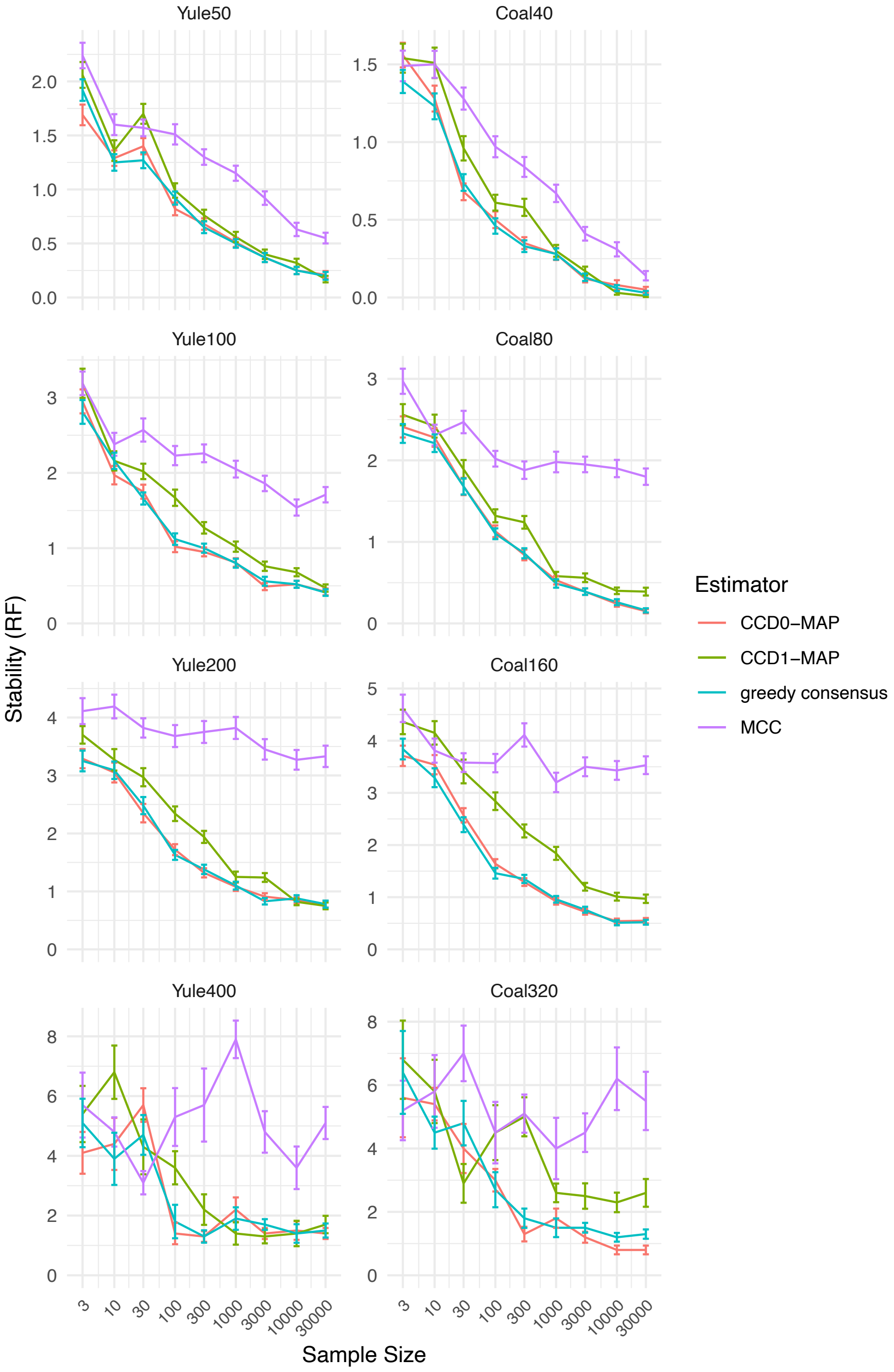

Fig N: The stability of the point estimates in terms of the RF distance, that is, the mean difference of RF distance of the point estimate to the truth of the two replicates of each simulation.

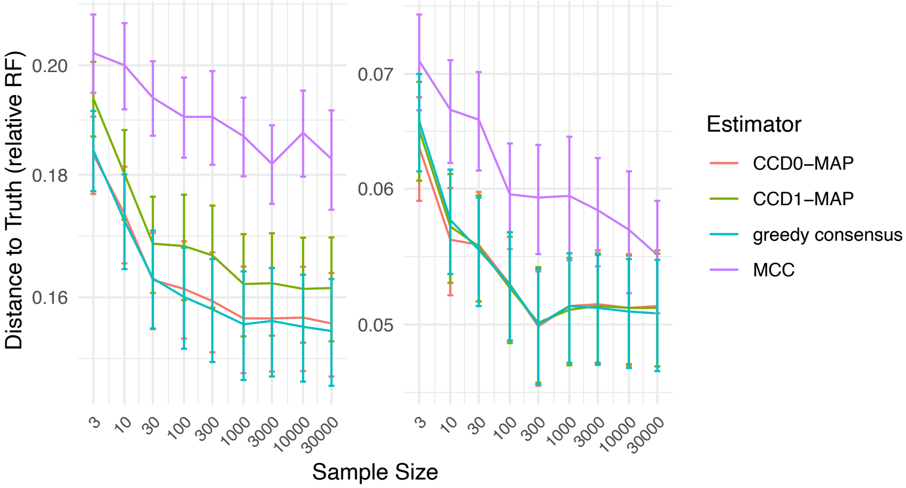

(a) Accuracy

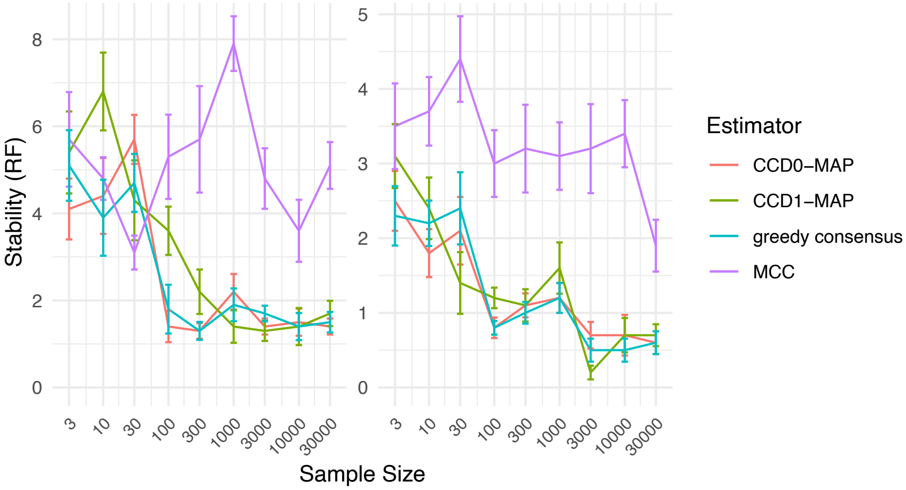

(b) Stability

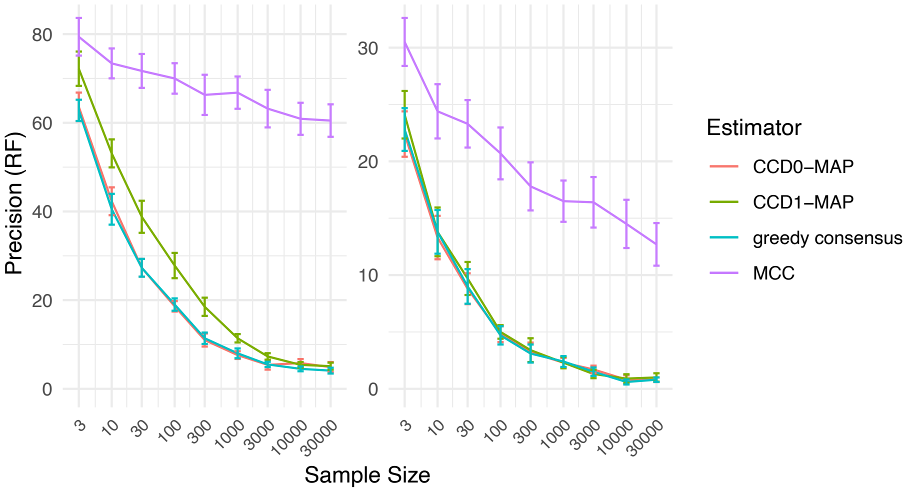

(c) Precision

Fig O: Comparing `Yule400` point estimates to `Yule400-Long`.
