# Supplementary material for: Accurate Bayesian phylogenetic point estimation using a tree distribution parameterized by clade probabilities
Source: PLoS Comput Biol. 2025 Feb 13;21(2):e1012789. doi: 10.1371/journal.pcbi.1012789 (PMC11835378; doi:10.1371/journal.pcbi.1012789)
Supplement: Attachment — Submitted filename: reviewsCCDPointEstimator.pdf [file pcbi.1012789.s002.pdf]

Response to reviews of "Accurate Bayesian phylogenetic point estimation using a tree distribution parameterized by clade probabilities"

June 2024

We would like to thank the three reviewers for their great comments and suggestions on how to improve the paper! We have addressed all comments by the reviewer either by making the suggested changes or by giving a response below (in blue). The major changes resulting from the comments are as follows:

- The new literature about the work by Matsen and colleagues is now incorporated. In particular, we added a new model to our distribution experiments, CCD2 which is equivalent to their sDAG model.
- As suggested, we looked at real datasets, namely DS1–4. We found that for these simple problems, the richer models (CCD0 $<$ CCD1 $<$ CCD2 $<$ sample) perform better.
- Based on these new observations, we changed the recommendation in the discussion and added some preliminary experiments to the appendix that look at AIC scores to pick the best model. However, a full investigation and solid recommendations for all types of datasets requires a lot more work that would go beyond the scope of this paper.
- As suggested, we considered the sample "MAP" tree. Although this is commonly referred to as the MAP tree, it is actually the tree topology of the sampled state that has the highest posterior *density*, which in general does not correspond to the tree topology with highest posterior support. We did not included it in the manuscript figures; see details in the respective comment below.
- As suggested, we've added a plot that considers the accuracy of the distributions in terms of clades (instead of trees).
- We now stress the difference between a parametrization of tree space and how to populate these parameters.

Response to Reviewers

Reviewer 1

The CCD0 described represents a substantial improvement in how we handle posterior samples in Bayesian phylogenetics. When it becomes adopted (which I am optimistic will happen soon) it will lead to an improvement in the generation of topology point estimates, (and despite being a technical manuscript will generate a large number of citations for PCB). I like this manuscript, the construction of the CCD0/1 are a very

good idea and will improve Bayesian phylogenetics. I want this published. However, I have several major comments I would like addressed before I would consider this ready for publication, in particular #1 and #2. Addressing them will require substantial revisions.

We have addressed all comments; see detailed responses below.

Major comments

1. The Remark, lines 190–195, is very concerning. If this is only an issue for CCD1, then that should be spelled out very clearly. If it is an issue for both CCD0 and CCD1 (which I think is the case) then this remark should be moved to a position where it is clearer that it applies to both and pointed out again in the Discussion. If you have a fool-proof remedy for this, I would really like to see that spelled out in detail. If you do not have a good remedy, then please remind the reader of this in the Discussion as it is something that must be kept in mind.

It is only an issue for CCD1, not for CCD0. Considering the example with a random tree T not sharing any non-trivial clade with the other $k-1$ trees, note that T would have probability only $(1/k)^{n-2}$.
In addition, we now mention a second idea which we have also implemented as a warning system in TreeAnnotator. However, there is no fool-proof remedy since it also depends a lot on the data. For very noisy data and few trees, it could just be that there are trees that do not share many clades with other trees.

2. Lines 203–227 are obviously important, and I think I understood them after a lot of effort (and frustration). I think the paper will be much improved if you (a) replace this section with a high level overview of the motivation for CCD0 and how it differs from CCD1; and (b) put this section into an appendix, and elaborate and simplify the derivation for struggling readers (such as myself). This way a reader can get a feel for the method (without being scared off) and readers interested in details can enjoy an expanded derivation in the appendix.

Done as suggested.

3. Figure 2 is very helpful, but I think you can help the reader by leaning on it a little more. For example, there is a lot of notation, you could give examples of a lot more of the notation by spelling it out with this example. Also, I think it will be easier to see the bipartite structure if you use more distinct colours in Figure 2.

Figure is referenced earlier now, colors added and figures merged.

4. Optional but important: To hasten uptake of CCD0 over MCC, please consider putting together a Taming the BEAST tutorial with a clean example.

We've updated the tutorials in LPhy and the tutorials we manage in Taming the Beast. We also contacted others responsible for Taming the Beast tutorials.

Minor comments

Figure 5 is great!

Thanks!

Line 45: “stickiness” seems a bit vague.

Rephrased

Line 49: I think it would be helpful to introduce the acronym ”MRC” here.

Done

Line 83: It might help to indicate if you intend here to give a formal definition of ”tractable tree distribution” here, or if this is just a short hand for a useful distribution.

Clarified (just a short hand)

Line 90: I disagree that other trees should *not* be represented here. I would have thought it was useful to have the trees assigned a very low probability so that I know it is accounted for by the method, i.e. it is known to be low probability, not an oversight. Perhaps you expand on this to explain your stance, am I missing something?

We have removed this sentence.

Line 173: I find the vertical bar to indicate a split to be distracting since it is close to conditioning when used within a probability, perhaps you could consider using hyphens instead.

Changed, we use $\|$ now.

Line 285: Notation is unpleasant in Equation (1) now Eq. 6 here please give extra consideration to hyphen or similar.

Here $|$ actually indicates conditionality.

Line 188–189: Possible inconsistency in the notation?

Fixed

Line 199: this sentence is a bit awkward and will be cleaner if you reorder some of the words.

Rephrased

Line 204: Consider pointing out that this is the power set.

Done

Line 257: Maybe include the base case for the recursive calculation of entropy too?

Done

Line 347: Six distributions? I got a bit muddled on this line, why six?

Line 489: This is a really impactful statement, it could probably be repeated more explicitly in the introduction.

Done

Line 517: "caution against using" not "caution of using", I'm not sure.

Fixed

Discussion: I think it should be reasonably straightforward to represent multifurcating trees. It might be worth pointing this out in the discussion, but obviously not necessary.

Comment added

References: Some of the preprint server references are missing DOIs, journal and article titles have incorrect capitalisation

Fixed

Appendix: I see there is a blog post on getting this method running efficiently, please consider putting a copy of this as an appendix if there is significant scholarly content there.

We decided to not add the content of the blog post to this paper as we think that the implementation details and notes on standard optimization techniques are better off there.

Reviewer 2

Summary

The authors have provided a new parameterization of distributions on tree probabilities (CCD0) as well as interesting simulations concerning the performance of the original conditional clade distribution framework. They also implement MAP point estimates for these distributions. They find that these outperform the very commonly used maximum clade credibility tree. The paper is interesting, well structured, executed, and written.

My number one issue with this paper is that it makes quite a sweeping recommendation using only simulated data, and easy simulated data for that matter. It's important to note that for the most part, the evaluation of these models doesn't have to happen on simulated data, and instead can happen on real data with all of the quirks of real data. Because the simulation framework is very simple and so it's not perhaps not so surprising that the simplest estimator, CCD0, performs well. However, I wouldn't feel very confident in extending these conclusions to real data. In our work, using real data with multimodal posteriors, we have found that an even more complex estimator than CCD1 is needed to accurately capture tree posterior distributions.

We added experiments on real datasets.

Speaking of which, we have developed a more general framework than CCD1, including regularization and unrooted trees. See Zhang, C., & Matsen, F. A., IV., *Generalizing Tree Probability Estimation via Bayesian Networks*, 2018. This work uses the terminology "subsplit" for "clade split".

Perhaps the most relevant part of this prior work is that we can think of these distributions as parameterizations of probability distributions on trees, and that we can use statistical methods to estimate parameters of these models. Specifically, one can use regularization to improve model fit. That idea doesn't seem to have made it into the current paper. We have also described how these models can be expressed as a graph: Jun et al. *A topology-marginal composite likelihood via a generalized phylogenetic pruning algorithm*, 2023.

> Thanks for making us aware of this work! We have added the related work throughout the paper. Specifically, we added sDAG in form of CCD2 to the methods and added more discussion on the topic of parametrization vs. estimating the parameters.

CCD0 is an interesting advance, specifically how it incorporates clade splits that don't exist in the original data set. However, it breaks some of the conditioning that can be useful. I wonder if there is something between CCD1 and CCD0, in which one could use the CCD1 conditional probability when that's possible, but uses the CCD0 probability when not. Or some balance between the two.

Comments

The introduction is written in a BEAST-centric way. I suggest that it would be worth noting that consensus trees have other applications than Bayesian point estimates.

> We made a few changes to address this.

L39: unrooted phylogenetic inferences don't have divergence times

> Rephrased

Fig 1 & 2: It would be nice to refer to combine figure 1 and figure 2 into a single figure and refer to them right away when introducing the forest network, rather than waiting until introducing the example. It'd be great to expand the figure legend to describe the symbology for $\mathcal{C}$ and $\mathcal{S}$.

> Done

L185: "Does removing a tree T from the CCD1 and thus decreasing clade and clade split frequencies by one, significantly change the probability of T." Can't we just look for edges of probability $1/k$?

> Indeed, we have added this comment to the paper and also implemented it as a warning system in TreeAnnotator based on this principle.

L203: It would be helpful to clarify the goal of this paragraph more precisely than "based on a function F".

Paragraph moved to appendix and rephrased for clarification

MRE: please specify what this means exactly

Done

Figure 5: please identify the labels S, CCD0, CCD1 in the figure legend.

Done

Why use a multiple of 100 for the Yule simulations, but not for the coal simulations?

We found that Coal40 was more comparable to Yule50 and based the multiples on that.

Reviewer 3

Major comments

My main comment regarding the manuscript is the omission of the MAP tree as a point estimator. Personally, I would always choose the MAP tree over the MCC or consensus tree, especially if those are restricted to correspond to a sampled tree. If I believe that my MCMC samples are representative of the posterior distribution, then why not choose the tree with the highest posterior probability? My suggestion is therefore to include the MAP tree in the evaluations (Fig 5). Would it be possible to include the MAP tree as an estimator in Figure 9? Or can you say how it would perform?

We had a look at the so-called "MAP" tree (i.e. the topology of the highest posterior *density* state from the sample) and plotted it against the other point estimates; we have added the plots below, see Figure 1. We found that it consistently performed worse than the MCC tree for these measurements and so decided to not include this point estimate in the paper. However, we now also discuss the MAD tree in a full paragraph in the discussion.

If I understood the results correctly, then the CCD0 and CCD1 provide better estimates of the posterior probability of the MAP tree (or true tree?) than the simple sample frequency from the Monte Carlo samples. If this is true, then I would conclude that it is always better to use the CCD0 or CCD1 to estimate the posterior probabilities of the MAP tree. Perhaps this could be emphasized?

Yes, in general it is better to use the CCDs to estimate the probability of any tree instead of the sample frequencies. Furthermore, if the goal is to find a point estimate, we generally recommend using the CCD0-MAP tree (or the greedy consensus tree). However, as we now mention in the paper, this recommendation depends on the specific dataset and should not be used blindly. On the DS1 for example it would be possible to use the CCD-MAP trees, but there is actually a

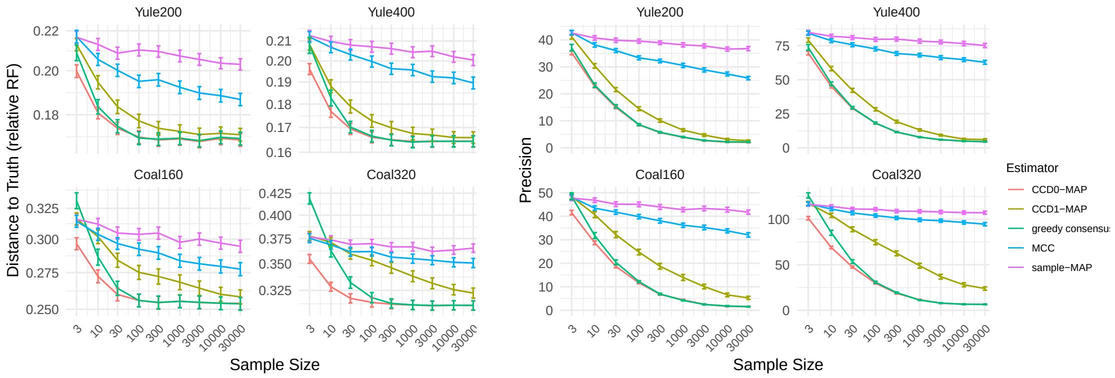

(a) The accuracy of the point estimates measured in terms of the mean relative RF distance to the true tree for different sample sizes of the large datasets.

(b) The precision of the point estimates in terms of the RF distance, that is, the mean RF distance of the point estimates of the two runs of each simulation.

Figure 1: Including the sample "MAP" tree for accuracy and precision. Although this is commonly referred to as the MAP tree, it is actually the tree topology of the sampled state that has the highest posterior *density*, which in general does not correspond to the tree topology with highest posterior support

tree within the posterior that has been sampled 11% of the time, hence on could also pick this highly probable tree. We have added this to the discussion, besides a general recommendation for CCD0 on harder datasets (in terms of high entropy), more work on picking the right CCD for any particular dataset is needed.

Perhaps you could add a small subsection about the evaluation of your simulations in the methods section, especially including an equation for the MAE. This is even more important for the MRE. How did you use the credible interval for these plots (Fig 6)?

We have added a sentence in the paper about how the MAE and the MRE are defined. We also added a sentence of how we calculate the credible set of trees.

Would it be possible to plot the actual MAE and not only the wins? You could keep the same x-axis as in Fig 5, and plot 3 lines (or 3-times 5 lines) with the MAE for each method. The winning estimator could still be pretty bad. Maybe this would be similar to Fig 7?

We added these plots (Fig. 12) showing the MAE in the appendix.

From Table 1 it seems that the posterior distribution is relatively flat. Would it be possible to perform an experiment with an increasing number of sites in the alignment for the Yule50 trees to see the impact of the number of trees in the CI on the recovery frequency of the true tree?

We added another figure (Fig. 23) where we compare the point estimates for

Yule400 and Yule400-long, which has 4x the sequence length. As expected, they all converge closer to the truth.

Similarly, do you think you could plot the posterior probability of the true tree vs frequency of the true tree being contained?

We are not sure what this would reveal. The probability varies a lot with different entropies and for high-entropy and non-spiky datasets the true tree gets almost never sampled (cf. Table 1).

How specifically did you treat unresolved nodes in the computation of the RF distance? I'm specifically wondering because some implementations would give the star tree a 0-distance, which means that any consensus method that is ultra conservative will be less penalized.

This is a fair concern and we now mention it where we recall RF. In our implementation the star tree would get a distance of half the max distance to the true tree. However, since in our experiments the greedy consensus method produced unresolved trees only a few times and only for small sample sizes (cf. Table 2), we decided not to penalize it. In general, one could use a penalty of 1 for each "missing" clade towards fully resolvedness.

I know this might be asking a bit much, but do you think it would be possible to evaluate the estimators on empirical datasets? For example, you could use the very long runs to obtain a "true" tree and then compare the RF distance between the different tree estimators.

As suggested we used very long chains on the DS1–DS4 datasets. We added the experiments in the appendix and include the finding in the discussion.

Could you add some recommendation about how many trees one would need to sample? Hopefully no one would sample fewer than 100 trees.

The standard recommendation for the minimum number of trees still holds (based on ESSs etc.). Additionally, we usually recommend no more than 10k trees, just because bigger tree sets are not comfortable to work with and 10k tends to be enough for estimating clade support and clade age distributions accurately for practical purposes. However, for estimating tree distribution for the purpose of point estimate, rogue detection, etc. it may well depend on the entropy of the set of trees. Intuitively, low entropy sets require fewer trees to cover all properties of the tree distribution, though how many exactly is out of scope for this paper and part of future research (which we already started).

Since you mentioned in line 294 that CCDs could be used to obtain posterior probabilities of clades (or split frequencies), would it be possible to evaluate how well the CCD's perform compared with standard sample frequencies? Given that many people use posterior probabilities of clades to assess support, it would be nice if CCD's are more robust than simple Monte Carlo frequencies.

We added a plot (Fig. 5 and in the appendix Fig. 14) on the accuracy of the distributions in terms of clade probabilities and extended the note on this in the methods.

Finally, I was wondering if the CCD0 and CCD1 would perform better than sample frequencies from independent MCMC samples. Maybe I missed this in the discussion/conclusion?

We showed that they perform better at estimating tree probabilities (in their respective regimes, cf. Section 3.1).

Minor comments

line 43: Please define BHV

Done

line 282: dynamic program?

Rephrased

line 286: Is the CCD-MAP the same as the MCC except using conditional clade probabilities? Maybe that could be explained in the text.

We say CCD[i]-MAP tree now as this might have caused some confusion. CCD0-MAP uses clade frequencies converted into CCPs, so it uses the same as MCC but is not restricted to the sample, which is mentioned in the next paragraph.

line 326f: 2 chains → 2 replicates (chains could also mean one cold and one heated chain, so better use replicates to avoid confusion).

Changed

Fig 5: Please specify S in the caption

Done

Fig 7: What is the golden mode tree?

Rephrased for clarification
