# Supplementary material for: Accurate Bayesian phylogenetic point estimation using a tree distribution parameterized by clade probabilities
Source: PLoS Comput Biol. 2025 Feb 13;21(2):e1012789. doi: 10.1371/journal.pcbi.1012789 (PMC11835378; doi:10.1371/journal.pcbi.1012789)
Supplement: Attachment — Submitted filename: responseCCDPointEstimator2.pdf [file pcbi.1012789.s003.pdf]

# Response to second-round reviews (August 2024) of "Accurate Bayesian phylogenetic point estimation using a tree distribution parameterized by clade probabilities"

Dear Natalia Komarova,

Thank you for editing our submission! Also thanks to the reviewers for reviewing our revised version!

We have identified two major concerns raised by the referees, which we believe should be addressed. The first concern is that of Reviewer 1, who requested that our experiments on real datasets be presented more prominently. The second concern is that of Reviewer 2, who requested us to extend our experiments on real datasets. In what follows, we will explain why we decided to not put more emphasis on real data experiments, mainly due to the impossibility of larger experiments that remain meaningful when estimating the ground truth probabilities of trees is the objective. We agree that this point was not made sufficiently clear in the previous revision, so we have added a detailed and clear explanation of this reasoning.

To put these concerns into the context of our paper let us summarize how they fit within the papers narrative. The two main contributions of this paper are (i) the introduction of CCD0, a new model to estimate a posterior tree distribution and (ii) the introduction of point estimates based on the different CCD models. To evaluate the performance of the different CCD models and point estimates, the *true* posterior distribution (tree probabilities) as well as the *true* tree down which the data has evolved are required.

If we consider the popular RSV2 dataset with 129 taxa, which is used in many BEAST tutorials, we find that a typical run takes less than 10 minutes and the resulting sample is sufficient for estimating continuous parameters, but not the *true* tree probabilities required for our CCD evaluation. Indeed, the entropy of this dataset is above 44 and the estimate of the most probable tree $T$ is around $10^{-11}$. So we would need a sample of size of at least $10^{13}$ for an ESS of 100. With a typical sample frequency of one sample a second, this would take over $\mathbf{300,000}$ **years**. If we were to follow Reviewer 2's suggestion to run the analysis for DS6 (entropy$\sim$22, probability of $T$ about $3^{-6}$) the analysis would take over 3 years.

We emphasize that both RSV2 and DS6 are extremely small datasets, compared to what is typically analysed in phylogenetic practise. In fact, the fundamental difficulty of sampling trees in the super-exponentially growing treespace is the very reason why we have models such as CCD0 in the first place (as discussed in the introduction). We addressed all the remaining concerns, as usual by either implementing the suggested changes or giving our response below (in blue).

Kind regards,
Alexei Drummond, on behalf of all authors

# Response to Reviewers

## Reviewer 1

**1.** I would like the application to real data to appear in more detail in the results section. This is important for conveying the limitations of the CCDs. My read on this is: for complex problems the sample based answer is preferable *if* you can get a sufficient number of posterior samples, *but* the CCDs work better if you are limited to a small sample. If this is the correct interpretation of your results, I think it needs to be spelled out very clearly in the Discussion and the Conclusion.

> You are correct with this interpretation, however, it is also the exact reason we decided to not include the DS1–DS4 application in the main paper. To quote our letter to the editor:
>
> > If we consider the popular RSV2 dataset with 129 taxa, which is used in many BEAST tutorials, we find that a typical run takes less than 10 minutes and the resulting sample is sufficient for estimating continuous parameters, but not the *true* tree probabilities required for our CCD evaluation. Indeed, the entropy of this dataset is above 44 and the estimate of the most probable tree $T$ is around $10^{-11}$. So we would need a sample of size of at least $10^{13}$ for an ESS of 100. With a typical sample frequency of one sample a second, this would take over **300,000 years**.
>
> As highlighted by the RSV2 example, it is impossible to obtain sufficient amount of samples for the estimation of tree probabilities and evaluating the CCD models. The reason that we are able to evaluate the CCD models on DS1–DS4 is that we are able to sample enough trees to get "true" tree probabilities from an MCMC sample in reasonable time. Typical phylogenetic datasets are more complex and/or larger than any DS or the RSV2 data. Hence, including real datasets into the paper would require a very broad range of data set applications and much more work on model selection techniques (necessary for evaluation in the absence of true tree probabilities). This is clearly out of scope for this paper and we purposefully leave these problems open for future research.

**2.** The application to real data isn't mentioned in the abstract or introduction, which seems odd. Please include a brief mention of these case studies in both the abstract and the introduction. It is a nice addition, you should point it out to people!

> As we have decided to not include the study on the real datasets in the main part, we actively decided to not mention them in the abstract and introduction.

Otherwise, the authors have satisfactorily addressed all my previous comments. Congratulations on a great manuscript!

> Thank you!

## Reviewer 2

**1.** One of the questions addressed by the manuscript is which model (here denoted CCDX) is most appropriate for phylogenetic posterior distributions. In my previous review, I suggested that the manuscript would be improved using an analysis using real data. The revised paper now has a limited analysis in an appendix. Out of the 8 DS data sets, the authors have done the first four, two of which (DS2 and DS3) have quite small posterior distributions (in Table 1 of Zhang and Matsen 2018, these are the only data sets that have $< 100$ trees in a sampled posterior, while the datasets 4 through 8 have at least 1000 trees in an equivalent sampled posterior, and two datasets have $>$ 10,000).

If we set aside DS2 and DS3, the results in Zhang and Matsen as well as in the current appendix are clear that the richer models are better. The manuscript describes this accurately, saying

> "On the real datasets DS1 and DS4, the richer models of CCD1 and CCD2 can capture the structure of the posterior better than a CCD0 even for small number of sampled trees."

However, I'm confused by the text that follows that sentence:

> "However, for non-trivial problems, sampling enough trees with MCMC to reach the regimes of CCD1, CCD2, or sample distributions is often not feasible."

This seems surprising because the advantage of the more complex models is evident with only 30 samples for the data sets with a reasonably wide posterior distribution. That observation seems at odds with the overall conclusion:

> "Hence, CCD0 offers the overall best posterior estimate for hard problems (in terms of entropy). This raises the question of how to select the best model for real datasets, in particular, when they are relatively simple (in terms of entropy)."

as well as this sentence in the Discussion:

> "First, CCD0 is best for few samples in terms of accuracy, precision, and stability, then CCD1 catches up and becomes the best method in the mid range, while CCD2 and the sample distribution require a huge number of sampled trees to become competitive."

Please help me understand how these statements are not contradictory.

In general, I continue to feel that a diverse collection of real datasets (using all of the DS data sets as a minimum, but I'm sure these authors have other interesting data sets at hand) is the right way to answer the question of the appropriateness of the tree model because at the end of the day we care about performance on real data. If the authors agree, I think it would make sense to expand the real data analysis and put it in the main text, rather than in an appendix.

We appreciate your concern on this matter, however, we believe there is a fundamental misconception here. As you point out DS2 and DS3 have very small posterior distributions, the same is true for DS1 and DS4 (small treespace and "few" sampled trees). This is the reason we included **only** these four data sets. With a typical sample frequency of one second per sample, an analysis that would return a sample with an ESS of 100 on DS6 (entropy∼22, probability of most probable tree $T$ roughly $3^{-6}$) would take over 3 years. This issue persists for all remaining DS datasets, where we run into computational limitations due to the size and complexity of the tree space and posterior. However, such an impossible to obtain analysis is crucial for the evaluation of the different CCD models.

As you point out, for DS1–DS4 the richer models are better. However, the richest model in this case is the MCMC sample and therefore any CCD model is essentially unnecessary. Overall, we have come to believe that DS1–DS4 fall into the category of trivial problems for MCMC sampling. Therefore, the overall conclusion of this paper remains, CCD0 is the best model for any non-trivial problem, shown by our simulations.

In addition, you raise the issue of model selection which is essential for any further comparison of CCDs on real data. Of course, in reality we expect there to be a spectrum of scenarios, like in Fig. 4, where each of the models (CCD0, CCD1, CCD2, and MCMC sample) is best for a particular dataset setup. However, establishing a method to select the specific regime in any real scenario requires significant effort across a wide range of datasets. As we point out in our paper:

> "However, picking the right CCD model for a particular dataset remains a tricky problem that requires further research."

We consider this out of scope for this paper and leave it for future research.

**2.** I do understand that for another part of the paper, namely the part that uses these models to find a consensus tree, it's convenient to have a known ground truth tree, motivating the use of simulated data. However, even for that application, it seems like one could use statistics such as the expected distance to the trees in the golden run posterior as a metric. I would hesitate to make such sweeping conclusions as the authors do in the conclusion without more investigation into real data sets, with their quirks and difficulties.

A CCD MAP tree is by definition a mode, and estimating a mean or median tree is a very different problem. Evaluating the CCD point estimates as mean or median trees also brings up several challenges as we have to decide on a distance measure and on a reference "truth". Such an evaluation transforms the problem into picking a treespace and finding a centroid, a geometric problem entirely independent of tree probabilities. Would our choice of the treespace correctly reflect the performance of the point estimates considered here? Treespace geometry has been proven to be complex and counter-intuitive before.
In short, we endeavour not to mix mode and centroid estimates in this paper.

Research into centroid estimates is however an important and interesting challenge, which we now feel very encouraged to tackle, and CCDs could be useful for this as well.

**3.** The authors state "CCD2 which is equivalent to their sDAG model." It is true that the structure of CCD2 is equivalent to the sDAG model, but the inferential methods differ. Zhang and Matsen (2018) show an improvement to inference when adding regularization, especially when data is sparse. At least this should be acknowledged.

We now emphasize that our approach relies solely on samples, unlike sDAGs, where different techniques were employed to populate the parameters. This is in addition to the pre-existing point mentioned at the end of the conclusion.

**4.** It would seem important to provide the "denominator" of Table 1, namely the number of trees contained in the CCD. Yes, as we get more samples we are more likely to get the true tree, but also the number of expressible trees is going up very quickly. Sorry to miss this on the first round.

We don't think that the size plays the important role, but the model. Note that the sampled trees are contained in the CCD2, are contained in the CCD1, are contained in the CCD0. So it is a question of how fast a particular model extrapolates and whether it includes the true tree before the next more complex model does.
We could have reported (independent of Table 1) on the sizes of the CCDs, but the CCDs grow of course much faster than the sample distribution and they are huge for Yule50. In addition, the number of trees also varies by magnitudes between the different simulations on the same number of taxa.

**5.** Can the authors clarify the motivation for using AIC in this context? It seems like an appropriate choice for settings where one can't have a true out-of-sample data set, but in this case one can generate additional data set just by running the chain again. I could be missing something about the intent here.

AIC was our first idea for model selection and cross-validation might be another good avenue; we would have liked to think about that first! Our intent with showing AIC scores in the appendix was only to point out that model selection methods should be investigated and that some preliminary experiments look promising. However, a full model selection study is not part of this paper.

**Detail A** I'm not sure if I agree with the idea that entropy quantifies whether a problem is "hard". I think that "hard" cases are ones with complex multimodal distributions. In contrast, the uniform distribution on trees would have maximum entropy but is very easy for MCMC to traverse and CCD0 would capture it well. Perhaps "diffuse" would be a better term?

Concerning the *semantics* of the term "hard", we want to point out (in line with our main argument on golden runs above) that when we say "hard", we mean that we cannot obtain enough samples to get good estimates of tree, clade, or clade split probabilities. The entropy is a very good indicator for that. While the

uniform distribution is easy to describe implicitly, and a simple traversal operator can work effectively, the number of clades, clade splits, and trees grows super-exponentially. As a result, a sample quickly becomes insufficient in providing reliable tree frequencies. For $n$ taxa, there are $(2n-3)!!$ trees and $2^n - 1$ different partitions of the root clade alone. So in that sense the uniform distribution is already impossible to estimate via samples for $n = 10$. In fact, CCDs (or samples) can only work because actual posterior distributions only contain a vanishingly small part of treespace even when they themselves have exponential size. Of course, multi-modality adds (in most cases) another dimension to hardness and there are more factors that make phylogenetic inference problems challenging.

**Detail B** Figure 3 is nice, though it would be worth explaining the visual notation you are using (sister clade) in the caption.

Done!

**Data availability** The data link provided does not describe including the XML files for the real data sets. If that's the case then please amend the description.

We added the XMLs and our sampled posterior distributions to the data repository and added this to the paper.
