# Supplementary material for: Accurate Bayesian phylogenetic point estimation using a tree distribution parameterized by clade probabilities
Source: PLoS Comput Biol. 2025 Feb 13;21(2):e1012789. doi: 10.1371/journal.pcbi.1012789 (PMC11835378; doi:10.1371/journal.pcbi.1012789)
Supplement: Attachment — Submitted filename: responseCCDPointEstimator3.pdf [file pcbi.1012789.s004.pdf]

Response to third-round reviews (December 2024) of "Accurate Bayesian phylogenetic point estimation using a tree distribution parameterized by clade probabilities"

Dear Natalia Komarova,

Thanks again for taking care of our submission!

We have carefully addressed Reviewer 2's concerns regarding our discussion and conclusions drawn from evaluations with simulated versus real datasets. To this end, we have added several clarifying paragraphs and sentences throughout the paper to provide a more nuanced context for our conclusions.

We have further merged subfigures into single figures, and changed the names of figures and table in the supplementary material as requested.

Kind regards,
Jonathan Klawitter, on behalf of all authors

Response to Reviewer 2

1. In their response the authors justify not focusing more on real data experiments by stating that (1.) the true posterior distribution is required for evaluating CCD models. The key fact to keep in mind is that the true underlying posterior is not available for simulated data, just like it's not available for real data. There is no difference there. Thus, this is not itself an argument for using simulated data sets.

> This is correct; we know neither the true simulated nor the true real posterior distributions. We thus focused on small simulated and real datasets where we can obtain a golden run of sufficient quality. For the evaluation of point estimates, we don't need golden runs and use the distance to true trees of simulations.

2. In their response the authors justify not focusing more on real data experiments by stating that (2.) evaluation on real data sets is impossible because it takes too long get accurate posterior tree estimates. There are two factors that determine the amount of time required to get a posterior sample: the number of taxa, and the shape of the posterior distribution. The number of taxa can be modulated for a real data set by subsetting, just like one can simulate data sets of different sizes.

> This is a really good idea to get more real datasets! We will use this in future projects, but for this manuscript decided to stick with the current datasets and instead adjust the conclusions.

3. If the shape of the posterior distribution differs between real and simulated data sets, this is a strong argument for including real data sets in the evaluation. The methods provided here are to mathematically describe the shape of the posterior, so if real data

is different than simulated data then we will be misled by choosing our methods using simulated data. As a metaphor in a simpler 1-D case, one might imagine that simulated data always looks Normal, whereas real data would take a variety of shapes.

As far as I can tell, the authors do not show that the shape of the posterior distributions for simulations are similar to the shape of posterior distributions for real data. It seems to me that the one available point of comparison we have, namely the relative ranking of the models, differs significantly between simulations and the real data analysis. This seems important, even if the authors dismiss their real data analysis as trivial.

There is nothing wrong with doing a simulation study! These can be quite valuable. However, in this context it seems appropriate to make it clear in statements of conclusion that there are signs in this paper and others that conclusions would differ for real data.

> Indeed, the differences between simulated and real data puts a small asterisk on a lot of work in our field! We hope that we rephrased the wording in the discussion to your satisfaction. The focus is now more on the results from the simulation, while stressing the differences for real data. We also added a paragraph to the conclusion on evaluations on simulated vs. real data.
>
> Apologies also for the miscomunication. We didn't mean to call the analysis of real data trivial, but only wanted to express that the datasets we can manage have low phylogenetic entropy making them "trivial" for samplers.

⋆ sDAG vs CCD2

> We also made a few small changes to the paragraph on sDAGs and CCD2s, stating more clearly that they only share the same underlying structure but otherwise differ.
>
> Thank you!
